# Supplementary material for: Anticancer Activity of Novel Difluorinated Curcumin Analog and Its Inclusion Complex with 2-Hydroxypropyl-β-Cyclodextrin against Pancreatic Cancer
Source: Int J Mol Sci. 2023 Mar 28;24(7):6336. doi: 10.3390/ijms24076336 (PMC10093935; doi:10.3390/ijms24076336)
Supplement: Supplementary file 1 [file ijms-24-06336-s001.zip › ijms-2156387-supplementary.pdf]

Legends to Supplementary Figures

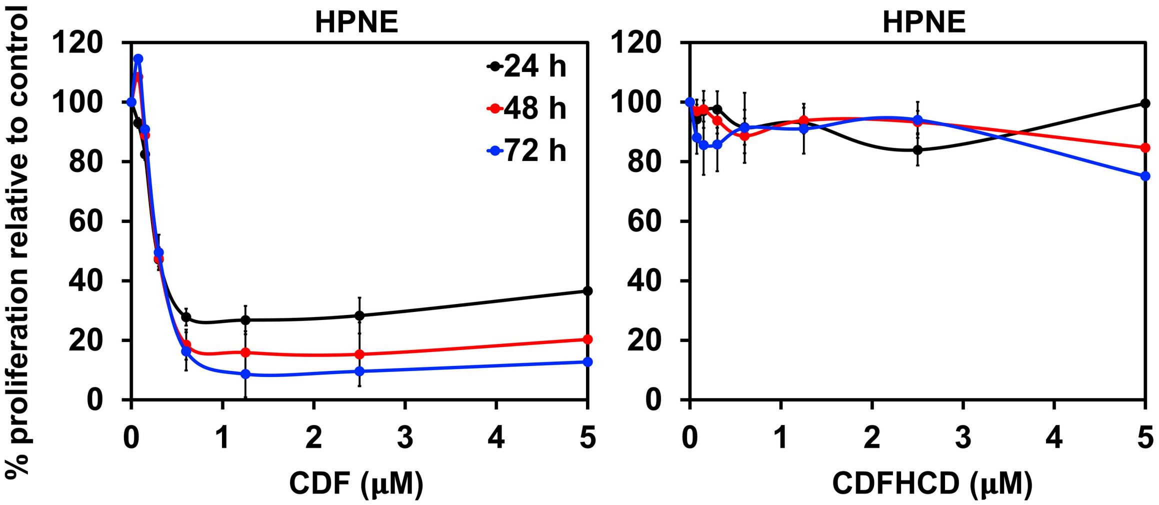

Supplementary Figure S1. CDF, but not CDFHCD, inhibits the immortalized pancreatic ductal epithelial cell line (HPNE) proliferation. HPNE cells were treated with increasing concentrations of CDF and CDFHCD (0–5 µM) and examined using hexosaminidase assay.

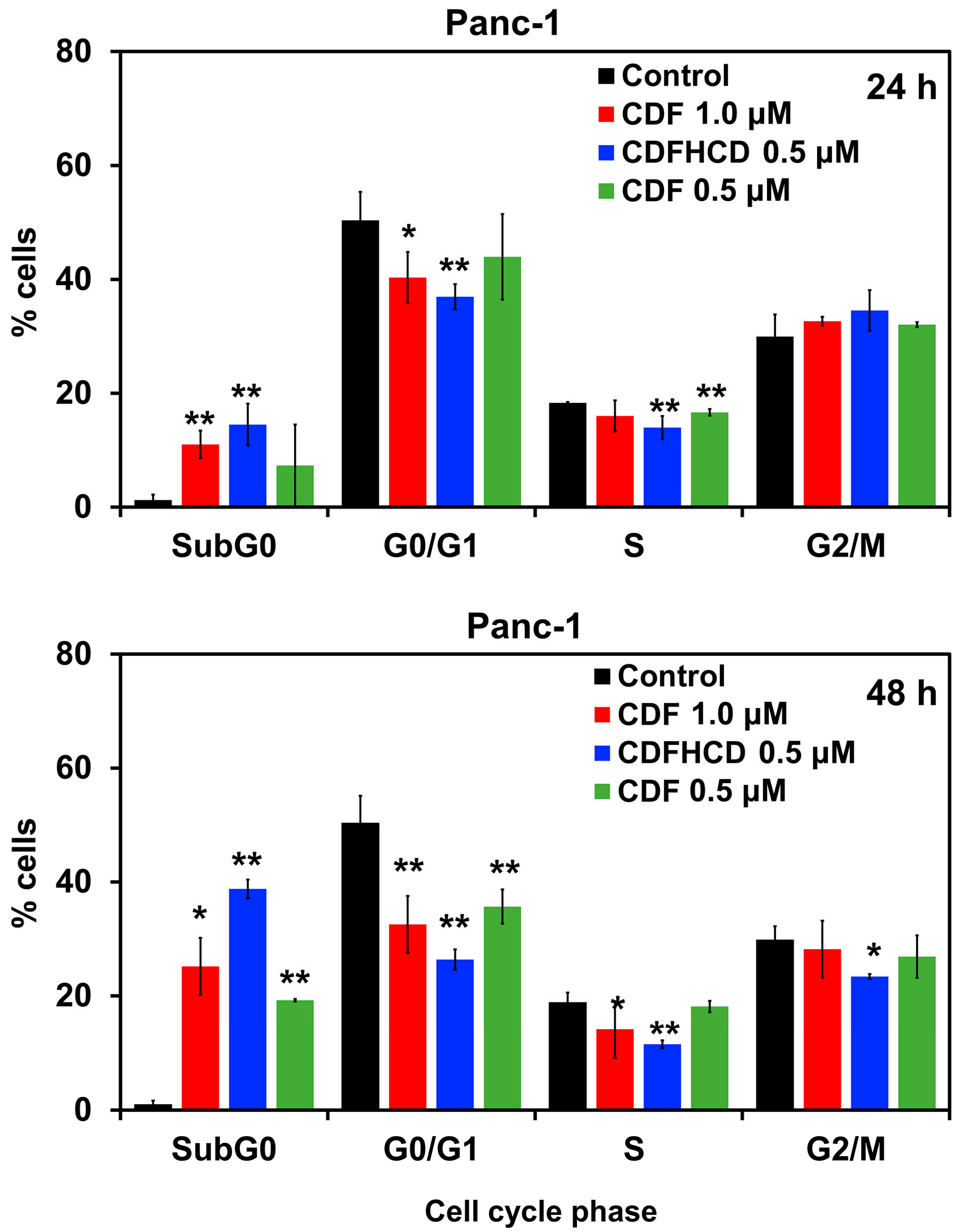
Panc-1
24 h
Control
CDF 1.0 μM
CDFHCD 0.5 μM
CDF 0.5 μM
% cells
80
60
40
20
0
SubG0
G0/G1
S
G2/M
Panc-1
48 h
Control
CDF 1.0 μM
CDFHCD 0.5 μM
CDF 0.5 μM
% cells
80
60
40
20
0
SubG0
G0/G1
S
G2/M
Cell cycle phase

(A)

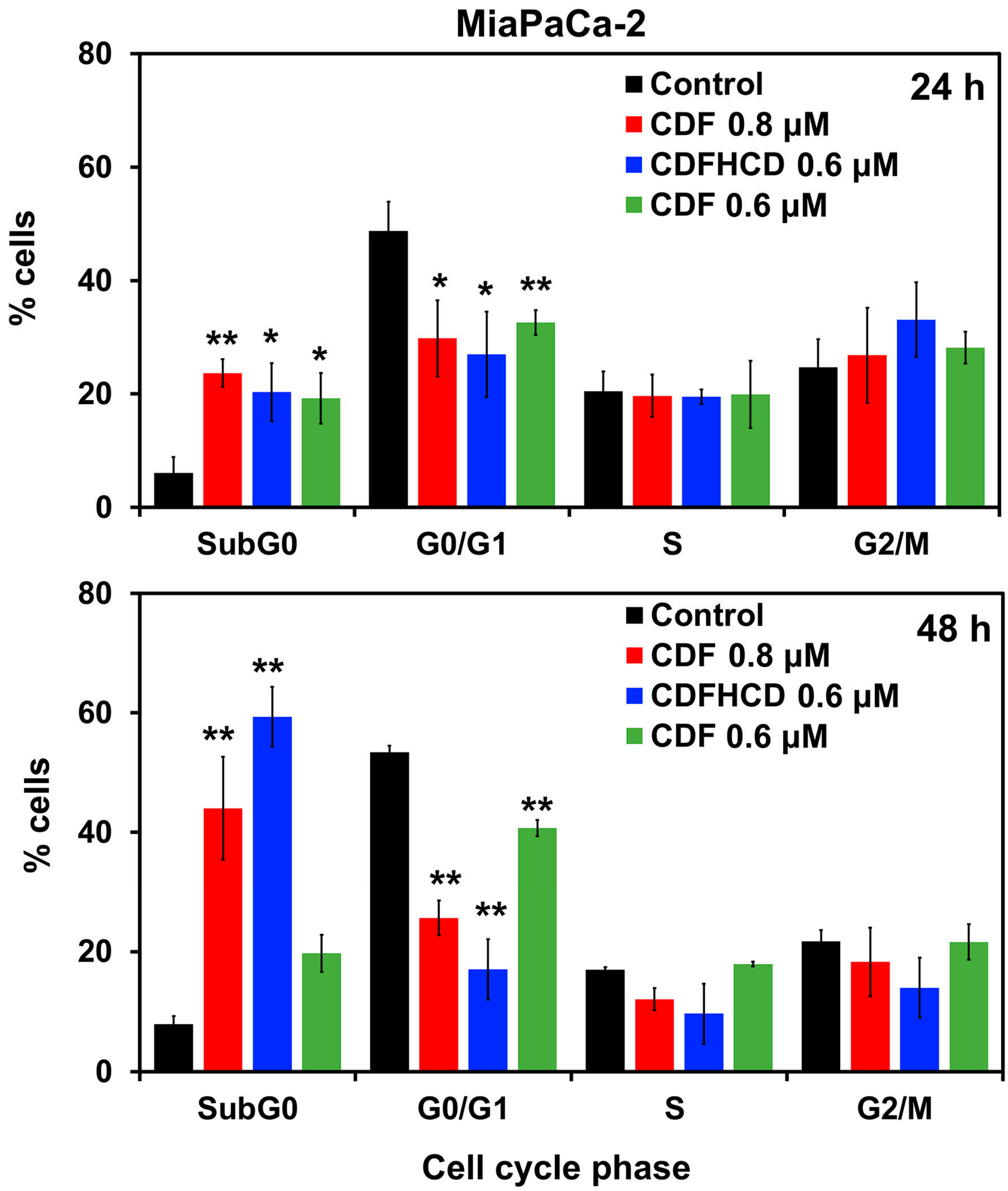

(B)

Supplementary Figure S2. Cell cycle analysis of CDF and CDFHCD in PDAC cells. (A) Panc-1 and **(B)** MiaPaCa-2 cells were treated with CDF and CDFHCD for 24 h and 48 h and analyzed by flow cytometry using FxCycle™ PI/RNase staining solution. The graph shows the percentage of cells in each cell cycle phase. (*$p < 0.05$, ** $p < 0.01$).

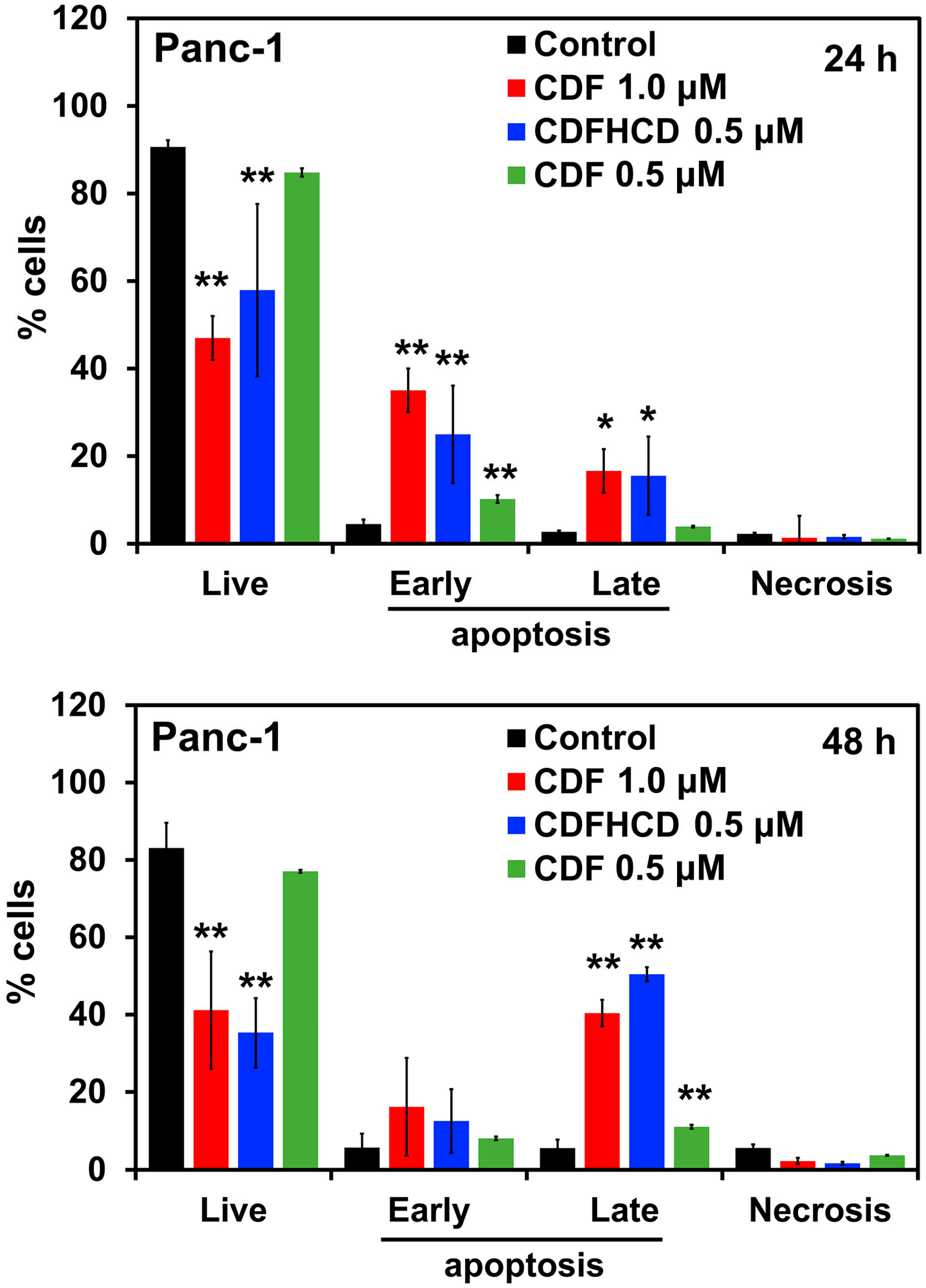
Panc-1
24 h
Control
CDF 1.0 μM
CDFHCD 0.5 μM
CDF 0.5 μM
% cells
0
20
40
60
80
100
120
**
*
Live
Early
Late
Necrosis
apoptosis
Panc-1
48 h
Control
CDF 1.0 μM
CDFHCD 0.5 μM
CDF 0.5 μM
% cells
0
20
40
60
80
100
120
**
Live
Early
Late
Necrosis
apoptosis

(A)

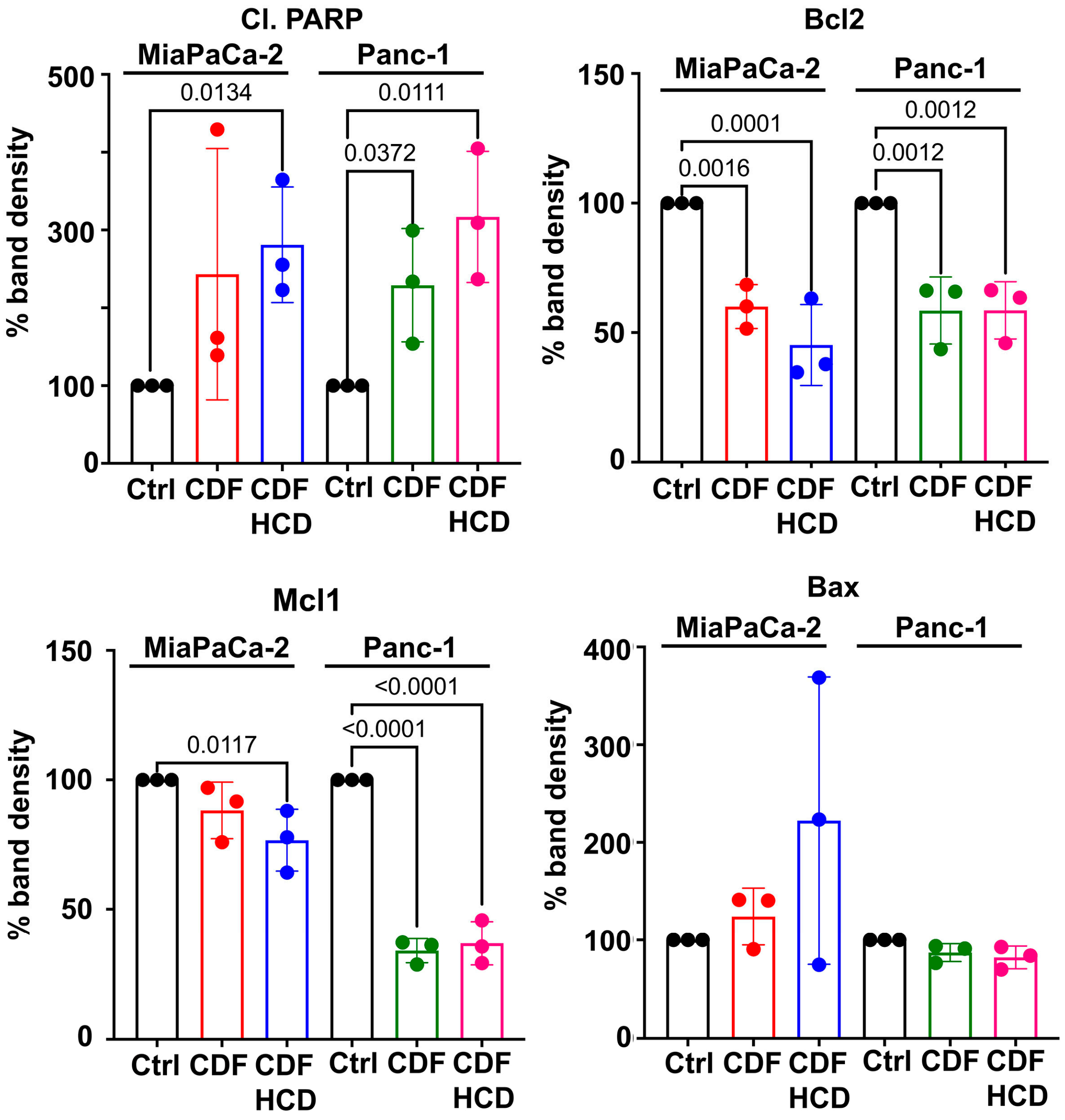

(B)

Supplementary Figure S3. CDF and CDFHCD induce apoptosis. (A). Panc-1 cells were treated with CDF and CDFHCD, stained with Annexin V-FITC and PI, and examined by flow cytometry. The graph shows the percentage of cells in apoptosis (early and late) and necrosis. (B) PDAC cell lysates treated with CDF and CDFHCD were studied by western blot to study the changes in apoptotic marker proteins, PARP, Mcl1, Bax, and Bcl2 expression. The graph shows the quantification of these protein levels. (*$p < 0.05$, ** $p < 0.01$).

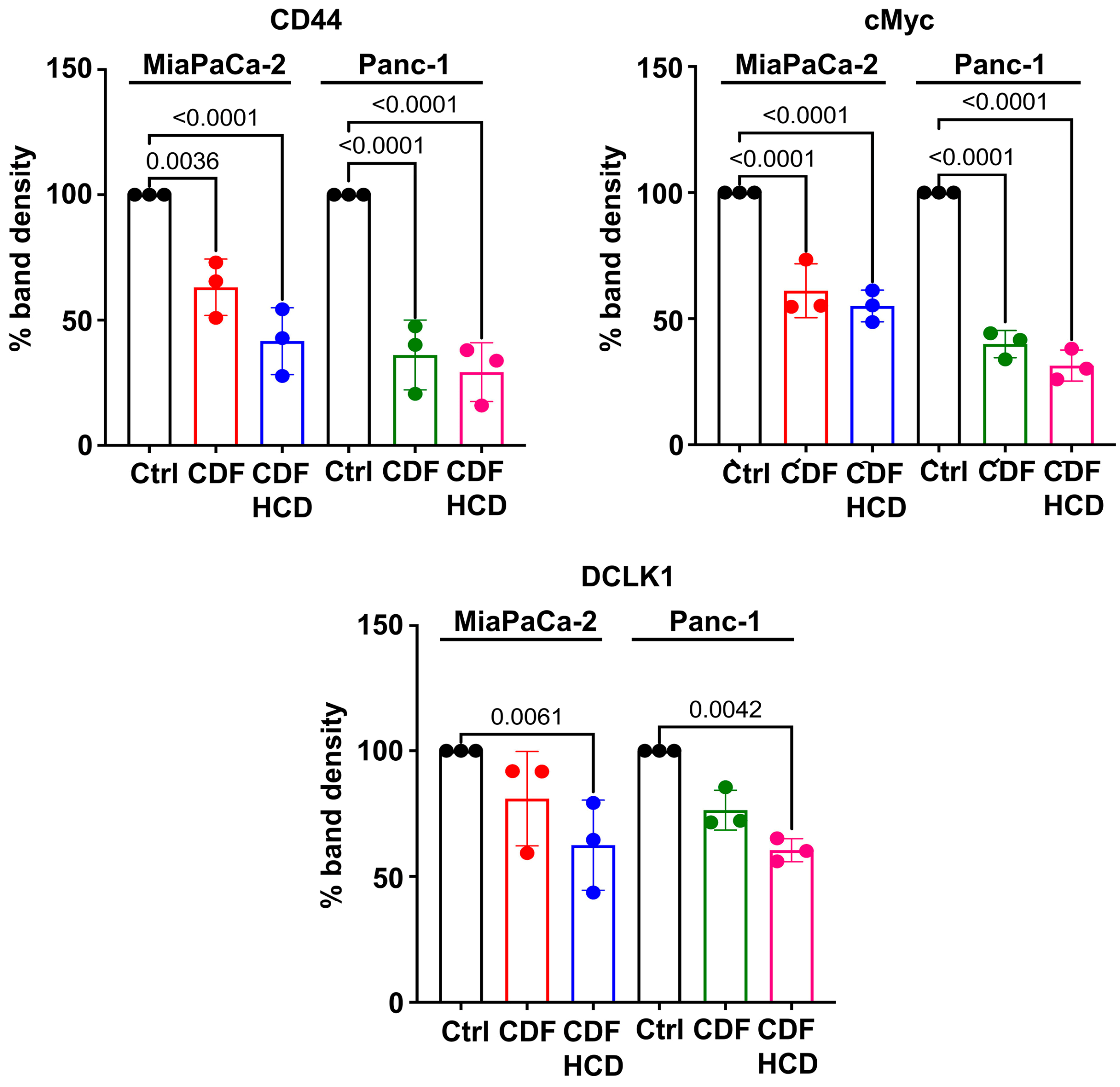

Supplementary Figure S4. CDF and CDFHCD inhibit spheroid formation. PDAC cell lysates treated with CDF and CDFHCD were analyzed by western blot to study the changes in CEC marker proteins, CD44, cMyc, and DCLK1 expression. The graph shows the quantification of these protein levels.
